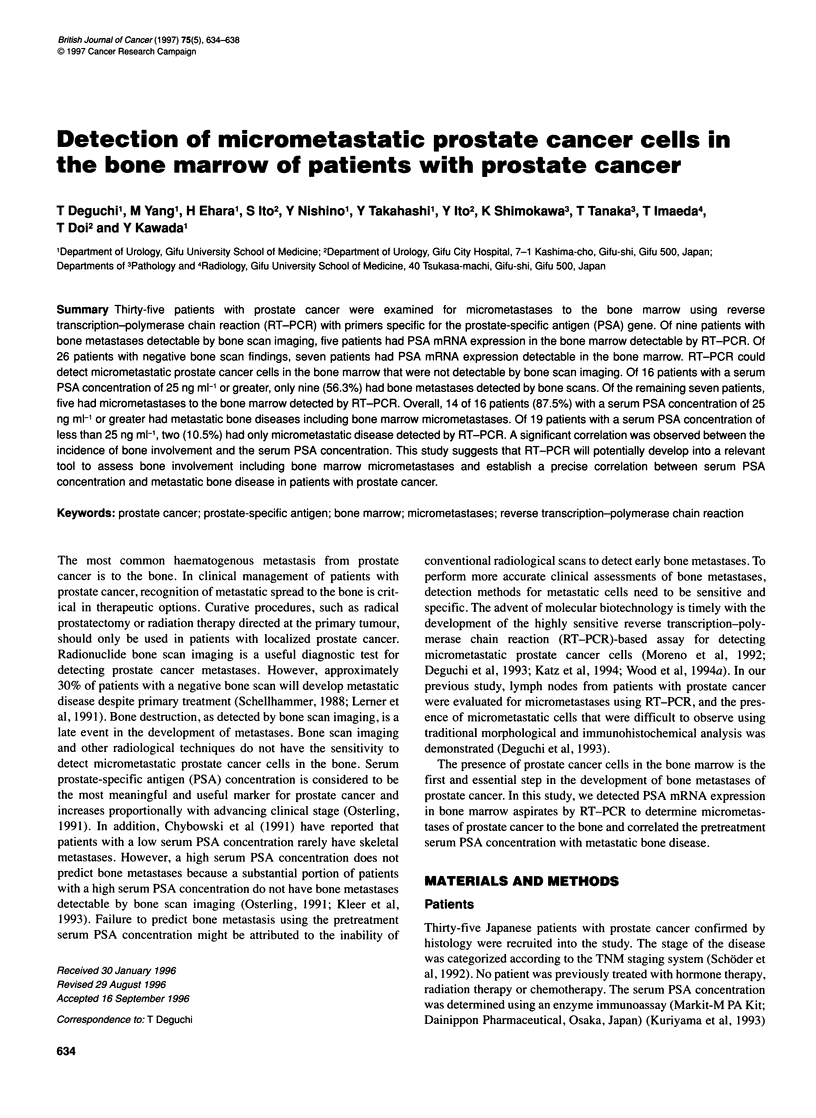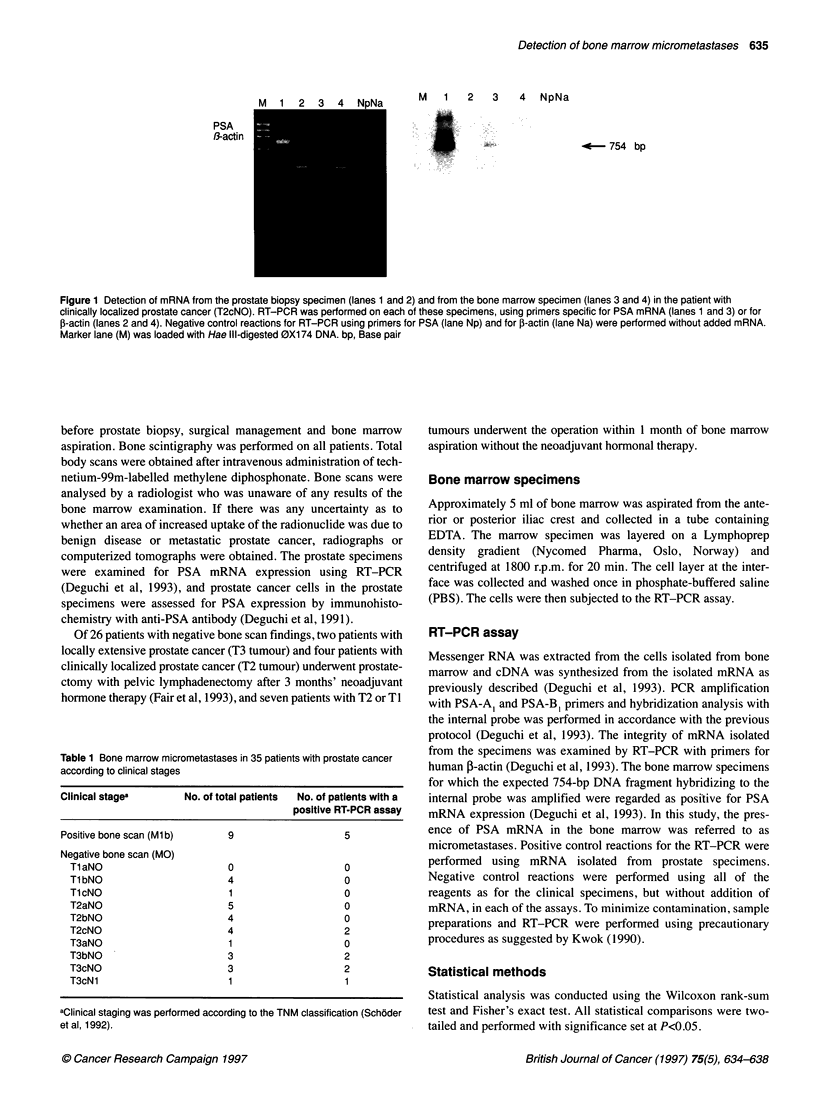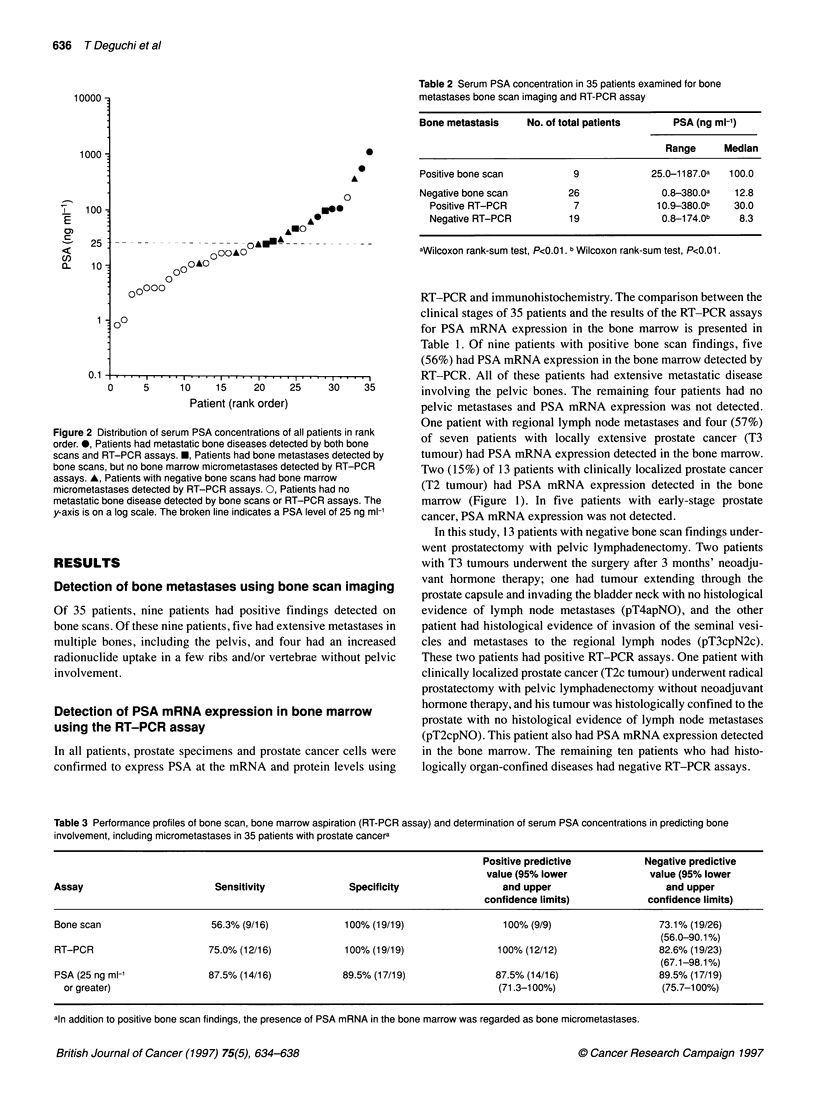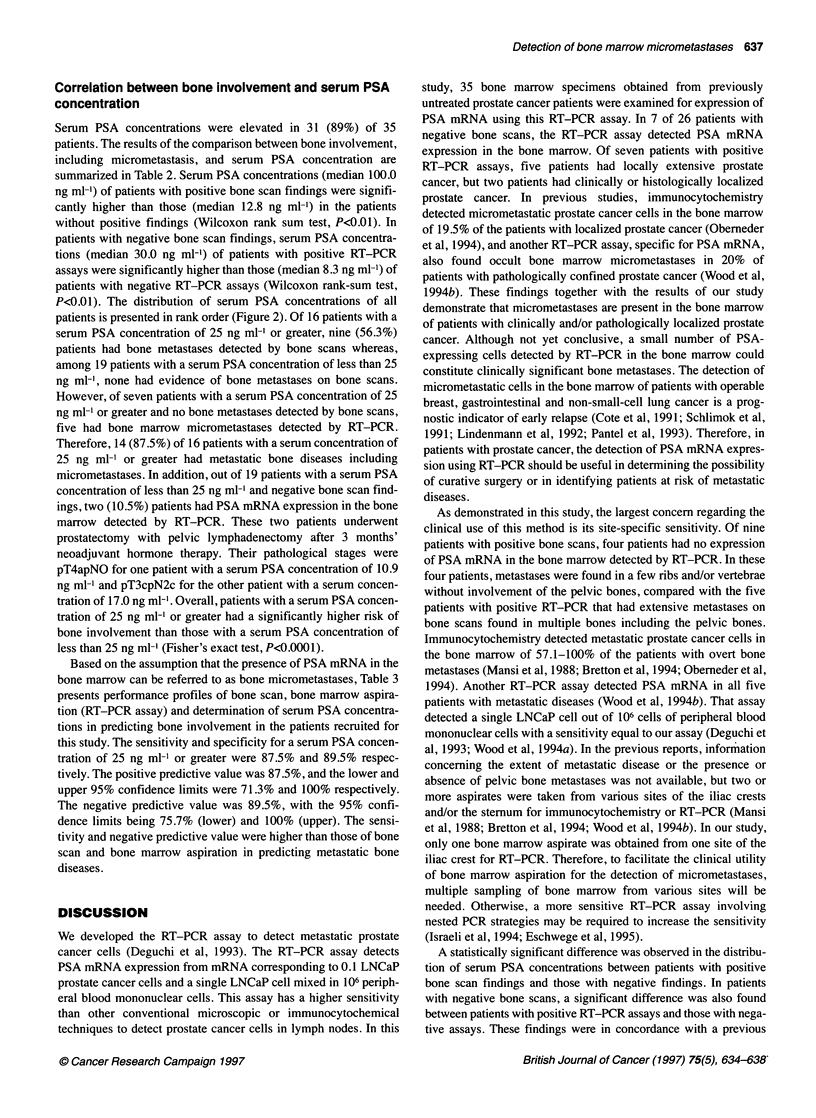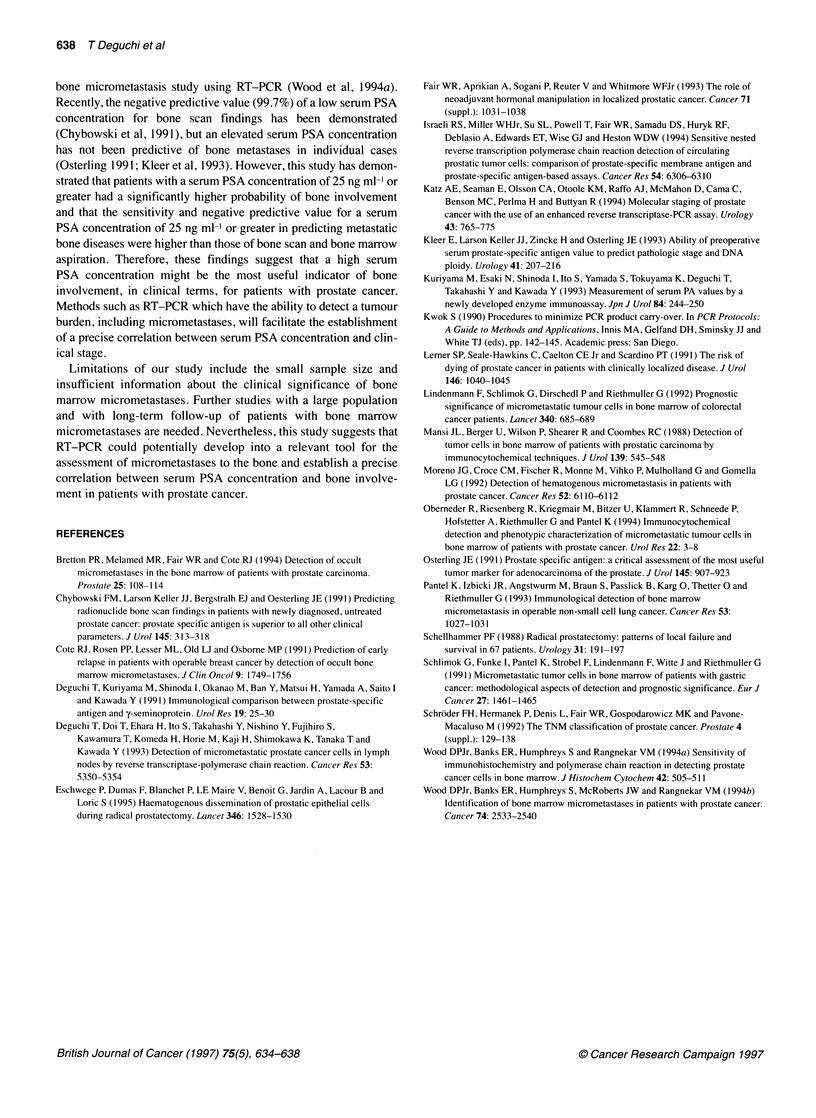# Detection of micrometastatic prostate cancer cells in the bone marrow of patients with prostate cancer.

**DOI:** 10.1038/bjc.1997.114

**Published:** 1997

**Authors:** T. Deguchi, M. Yang, H. Ehara, S. Ito, Y. Nishino, Y. Takahashi, Y. Ito, K. Shimokawa, T. Tanaka, T. Imaeda, T. Doi, Y. Kawada

**Affiliations:** Department of Urology, Gifu University School of Medicine, Gifu-shi, Japan.

## Abstract

**Images:**


British Journal of Cancer (1997) 75(5), 634–638
© 1997 Cancer Research Campaign

### Detection of micrometastatic prostate cancer cells in the bone marrow of patients with prostate cancer

T Deguchi[1], M Yang[1], H Ehara[1], S Ito[2], Y Nishino[1], Y Takahashi[1], Y Ito[2], K Shimokawa[3], T Tanaka[3], T Imaeda[4], T Doi[2] and Y Kawada[1]

[1]Department of Urology, Gifu University School of Medicine; [2]Department of Urology, Gifu City Hospital, 7–1 Kashima-cho, Gifu-shi, Gifu 500, Japan; Departments of [3]Pathology and [4]Radiology, Gifu University School of Medicine, 40 Tsukasa-machi, Gifu-shi, Gifu 500, Japan

**Summary** Thirty-five patients with prostate cancer were examined for micrometastases to the bone marrow using reverse transcription–polymerase chain reaction (RT–PCR) with primers specific for the prostate-specific antigen (PSA) gene. Of nine patients with bone metastases detectable by bone scan imaging, five patients had PSA mRNA expression in the bone marrow detectable by RT–PCR. Of 26 patients with negative bone scan findings, seven patients had PSA mRNA expression detectable in the bone marrow. RT–PCR could detect micrometastatic prostate cancer cells in the bone marrow that were not detectable by bone scan imaging. Of 16 patients with a serum PSA concentration of 25 ng $ml^{-1}$ or greater, only nine (56.3%) had bone metastases detected by bone scans. Of the remaining seven patients, five had micrometastases to the bone marrow detected by RT–PCR. Overall, 14 of 16 patients (87.5%) with a serum PSA concentration of 25 ng $ml^{-1}$ or greater had metastatic bone diseases including bone marrow micrometastases. Of 19 patients with a serum PSA concentration of less than 25 ng $ml^{-1}$, two (10.5%) had only micrometastatic disease detected by RT–PCR. A significant correlation was observed between the incidence of bone involvement and the serum PSA concentration. This study suggests that RT–PCR will potentially develop into a relevant tool to assess bone involvement including bone marrow micrometastases and establish a precise correlation between serum PSA concentration and metastatic bone disease in patients with prostate cancer.

**Keywords:** prostate cancer; prostate-specific antigen; bone marrow; micrometastases; reverse transcription–polymerase chain reaction

The most common haematogenous metastasis from prostate cancer is to the bone. In clinical management of patients with prostate cancer, recognition of metastatic spread to the bone is critical in therapeutic options. Curative procedures, such as radical prostatectomy or radiation therapy directed at the primary tumour, should only be used in patients with localized prostate cancer. Radionuclide bone scan imaging is a useful diagnostic test for detecting prostate cancer metastases. However, approximately 30% of patients with a negative bone scan will develop metastatic disease despite primary treatment (Schellhammer, 1988; Lerner et al, 1991). Bone destruction, as detected by bone scan imaging, is a late event in the development of metastases. Bone scan imaging and other radiological techniques do not have the sensitivity to detect micrometastatic prostate cancer cells in the bone. Serum prostate-specific antigen (PSA) concentration is considered to be the most meaningful and useful marker for prostate cancer and increases proportionally with advancing clinical stage (Osterling, 1991). In addition, Chybowski et al (1991) have reported that patients with a low serum PSA concentration rarely have skeletal metastases. However, a high serum PSA concentration does not predict bone metastases because a substantial portion of patients with a high serum PSA concentration do not have bone metastases detectable by bone scan imaging (Osterling, 1991; Kleer et al, 1993). Failure to predict bone metastasis using the pretreatment serum PSA concentration might be attributed to the inability of conventional radiological scans to detect early bone metastases. To perform more accurate clinical assessments of bone metastases, detection methods for metastatic cells need to be sensitive and specific. The advent of molecular biotechnology is timely with the development of the highly sensitive reverse transcription–polymerase chain reaction (RT–PCR)-based assay for detecting micrometastatic prostate cancer cells (Moreno et al, 1992; Deguchi et al, 1993; Katz et al, 1994; Wood et al, 1994*a*). In our previous study, lymph nodes from patients with prostate cancer were evaluated for micrometastases using RT–PCR, and the presence of micrometastatic cells that were difficult to observe using traditional morphological and immunohistochemical analysis was demonstrated (Deguchi et al, 1993).

The presence of prostate cancer cells in the bone marrow is the first and essential step in the development of bone metastases of prostate cancer. In this study, we detected PSA mRNA expression in bone marrow aspirates by RT–PCR to determine micrometastases of prostate cancer to the bone and correlated the pretreatment serum PSA concentration with metastatic bone disease.

### MATERIALS AND METHODS

### Patients

Thirty-five Japanese patients with prostate cancer confirmed by histology were recruited into the study. The stage of the disease was categorized according to the TNM staging system (Schöder et al, 1992). No patient was previously treated with hormone therapy, radiation therapy or chemotherapy. The serum PSA concentration was determined using an enzyme immunoassay (Markit-M PA Kit; Dainippon Pharmaceutical, Osaka, Japan) (Kuriyama et al, 1993)

*Received 30 January 1996*
*Revised 29 August 1996*
*Accepted 16 September 1996*

*Correspondence to:* T Deguchi

**Figure 1** Detection of mRNA from the prostate biopsy specimen (lanes 1 and 2) and from the bone marrow specimen (lanes 3 and 4) in the patient with clinically localized prostate cancer (T2cNO). RT–PCR was performed on each of these specimens, using primers specific for PSA mRNA (lanes 1 and 3) or for β-actin (lanes 2 and 4). Negative control reactions for RT–PCR using primers for PSA (lane Np) and for β-actin (lane Na) were performed without added mRNA. Marker lane (M) was loaded with *Hae* III-digested ØX174 DNA. bp, Base pair before prostate biopsy, surgical management and bone marrow aspiration. Bone scintigraphy was performed on all patients. Total body scans were obtained after intravenous administration of technetium-99m-labelled methylene diphosphonate. Bone scans were analysed by a radiologist who was unaware of any results of the bone marrow examination. If there was any uncertainty as to whether an area of increased uptake of the radionuclide was due to benign disease or metastatic prostate cancer, radiographs or computerized tomographs were obtained. The prostate specimens were examined for PSA mRNA expression using RT–PCR (Deguchi et al, 1993), and prostate cancer cells in the prostate specimens were assessed for PSA expression by immunohistochemistry with anti-PSA antibody (Deguchi et al, 1991).

Of 26 patients with negative bone scan findings, two patients with locally extensive prostate cancer (T3 tumour) and four patients with clinically localized prostate cancer (T2 tumour) underwent prostatectomy with pelvic lymphadenectomy after 3 months' neoadjuvant hormone therapy (Fair et al, 1993), and seven patients with T2 or T1 tumours underwent the operation within 1 month of bone marrow aspiration without the neoadjuvant hormonal therapy.

**Table 1** Bone marrow micrometastases in 35 patients with prostate cancer according to clinical stages

| Clinical stage[a] | No. of total patients | No. of patients with a positive RT-PCR assay |
|---|---|---|
| Positive bone scan (M1b) | 9 | 5 |
| Negative bone scan (MO) | | |
| T1aNO | 0 | 0 |
| T1bNO | 4 | 0 |
| T1cNO | 1 | 0 |
| T2aNO | 5 | 0 |
| T2bNO | 4 | 0 |
| T2cNO | 4 | 2 |
| T3aNO | 1 | 0 |
| T3bNO | 3 | 2 |
| T3cNO | 3 | 2 |
| T3cN1 | 1 | 1 |

[a]Clinical staging was performed according to the TNM classification (Schöder et al, 1992).

### Bone marrow specimens

Approximately 5 ml of bone marrow was aspirated from the anterior or posterior iliac crest and collected in a tube containing EDTA. The marrow specimen was layered on a Lymphoprep density gradient (Nycomed Pharma, Oslo, Norway) and centrifuged at 1800 r.p.m. for 20 min. The cell layer at the interface was collected and washed once in phosphate-buffered saline (PBS). The cells were then subjected to the RT–PCR assay.

### RT–PCR assay

Messenger RNA was extracted from the cells isolated from bone marrow and cDNA was synthesized from the isolated mRNA as previously described (Deguchi et al, 1993). PCR amplification with PSA-$A_1$ and PSA-$B_1$ primers and hybridization analysis with the internal probe was performed in accordance with the previous protocol (Deguchi et al, 1993). The integrity of mRNA isolated from the specimens was examined by RT–PCR with primers for human β-actin (Deguchi et al, 1993). The bone marrow specimens for which the expected 754-bp DNA fragment hybridizing to the internal probe was amplified were regarded as positive for PSA mRNA expression (Deguchi et al, 1993). In this study, the presence of PSA mRNA in the bone marrow was referred to as micrometastases. Positive control reactions for the RT–PCR were performed using mRNA isolated from prostate specimens. Negative control reactions were performed using all of the reagents as for the clinical specimens, but without addition of mRNA, in each of the assays. To minimize contamination, sample preparations and RT–PCR were performed using precautionary procedures as suggested by Kwok (1990).

### Statistical methods

Statistical analysis was conducted using the Wilcoxon rank-sum test and Fisher's exact test. All statistical comparisons were two-tailed and performed with significance set at $P<0.05$.

Figure 2 Distribution of serum PSA concentrations of all patients in rank order. ●, Patients had metastatic bone diseases detected by both bone scans and RT–PCR assays. ■, Patients had bone metastases detected by bone scans, but no bone marrow micrometastases detected by RT–PCR assays. ▲, Patients with negative bone scans had bone marrow micrometastases detected by RT–PCR assays. ○, Patients had no metastatic bone disease detected by bone scans or RT–PCR assays. The *y*-axis is on a log scale. The broken line indicates a PSA level of 25 ng $ml^{-1}$

### RESULTS

### Detection of bone metastases using bone scan imaging

Of 35 patients, nine patients had positive findings detected on bone scans. Of these nine patients, five had extensive metastases in multiple bones, including the pelvis, and four had an increased radionuclide uptake in a few ribs and/or vertebrae without pelvic involvement.

### Detection of PSA mRNA expression in bone marrow using the RT–PCR assay

In all patients, prostate specimens and prostate cancer cells were confirmed to express PSA at the mRNA and protein levels using RT–PCR and immunohistochemistry. The comparison between the clinical stages of 35 patients and the results of the RT–PCR assays for PSA mRNA expression in the bone marrow is presented in Table 1. Of nine patients with positive bone scan findings, five (56%) had PSA mRNA expression in the bone marrow detected by RT–PCR. All of these patients had extensive metastatic disease involving the pelvic bones. The remaining four patients had no pelvic metastases and PSA mRNA expression was not detected. One patient with regional lymph node metastases and four (57%) of seven patients with locally extensive prostate cancer (T3 tumour) had PSA mRNA expression detected in the bone marrow. Two (15%) of 13 patients with clinically localized prostate cancer (T2 tumour) had PSA mRNA expression detected in the bone marrow (Figure 1). In five patients with early-stage prostate cancer, PSA mRNA expression was not detected.

In this study, 13 patients with negative bone scan findings underwent prostatectomy with pelvic lymphadenectomy. Two patients with T3 tumours underwent the surgery after 3 months' neoadjuvant hormone therapy; one had tumour extending through the prostate capsule and invading the bladder neck with no histological evidence of lymph node metastases (pT4apNO), and the other patient had histological evidence of invasion of the seminal vesicles and metastases to the regional lymph nodes (pT3cpN2c). These two patients had positive RT–PCR assays. One patient with clinically localized prostate cancer (T2c tumour) underwent radical prostatectomy with pelvic lymphadenectomy without neoadjuvant hormone therapy, and his tumour was histologically confined to the prostate with no histological evidence of lymph node metastases (pT2cpNO). This patient also had PSA mRNA expression detected in the bone marrow. The remaining ten patients who had histologically organ-confined diseases had negative RT–PCR assays.

Table 2 Serum PSA concentration in 35 patients examined for bone metastases bone scan imaging and RT-PCR assay

| Bone metastasis | No. of total patients | PSA (ng $ml^{-1}$) | |
|---|---|---|---|
| | | Range | Median |
| Positive bone scan | 9 | 25.0–1187.0[a] | 100.0 |
| Negative bone scan | 26 | 0.8–380.0[a] | 12.8 |
| Positive RT-PCR | 7 | 10.9–380.0[b] | 30.0 |
| Negative RT-PCR | 19 | 0.8–174.0[b] | 8.3 |

[a]Wilcoxon rank-sum test, $P<0.01$. [b] Wilcoxon rank-sum test, $P<0.01$.

Table 3 Performance profiles of bone scan, bone marrow aspiration (RT-PCR assay) and determination of serum PSA concentrations in predicting bone involvement, including micrometastases in 35 patients with prostate cancer[a]

| Assay | Sensitivity | Specificity | Positive predictive value (95% lower and upper confidence limits) | Negative predictive value (95% lower and upper confidence limits) |
|---|---|---|---|---|
| Bone scan | 56.3% (9/16) | 100% (19/19) | 100% (9/9) | 73.1% (19/26) (56.0–90.1%) |
| RT–PCR | 75.0% (12/16) | 100% (19/19) | 100% (12/12) | 82.6% (19/23) (67.1–98.1%) |
| PSA (25 ng $ml^{-1}$ or greater) | 87.5% (14/16) | 89.5% (17/19) | 87.5% (14/16) (71.3–100%) | 89.5% (17/19) (75.7–100%) |

[a]In addition to positive bone scan findings, the presence of PSA mRNA in the bone marrow was regarded as bone micrometastases.

© Cancer Research Campaign 1997

### Correlation between bone involvement and serum PSA concentration

Serum PSA concentrations were elevated in 31 (89%) of 35 patients. The results of the comparison between bone involvement, including micrometastasis, and serum PSA concentration are summarized in Table 2. Serum PSA concentrations (median 100.0 ng $ml^{-1}$) of patients with positive bone scan findings were significantly higher than those (median 12.8 ng $ml^{-1}$) in the patients without positive findings (Wilcoxon rank sum test, $P<0.01$). In patients with negative bone scan findings, serum PSA concentrations (median 30.0 ng $ml^{-1}$) of patients with positive RT–PCR assays were significantly higher than those (median 8.3 ng $ml^{-1}$) of patients with negative RT–PCR assays (Wilcoxon rank-sum test, $P<0.01$). The distribution of serum PSA concentrations of all patients is presented in rank order (Figure 2). Of 16 patients with a serum PSA concentration of 25 ng $ml^{-1}$ or greater, nine (56.3%) patients had bone metastases detected by bone scans whereas, among 19 patients with a serum PSA concentration of less than 25 ng $ml^{-1}$, none had evidence of bone metastases on bone scans. However, of seven patients with a serum PSA concentration of 25 ng $ml^{-1}$ or greater and no bone metastases detected by bone scans, five had bone marrow micrometastases detected by RT–PCR. Therefore, 14 (87.5%) of 16 patients with a serum concentration of 25 ng $ml^{-1}$ or greater had metastatic bone diseases including micrometastases. In addition, out of 19 patients with a serum PSA concentration of less than 25 ng $ml^{-1}$ and negative bone scan findings, two (10.5%) patients had PSA mRNA expression in the bone marrow detected by RT–PCR. These two patients underwent prostatectomy with pelvic lymphadenectomy after 3 months' neoadjuvant hormone therapy. Their pathological stages were pT4apNO for one patient with a serum PSA concentration of 10.9 ng $ml^{-1}$ and pT3cpN2c for the other patient with a serum concentration of 17.0 ng $ml^{-1}$. Overall, patients with a serum PSA concentration of 25 ng $ml^{-1}$ or greater had a significantly higher risk of bone involvement than those with a serum PSA concentration of less than 25 ng $ml^{-1}$ (Fisher's exact test, $P<0.0001$).

Based on the assumption that the presence of PSA mRNA in the bone marrow can be referred to as bone micrometastases, Table 3 presents performance profiles of bone scan, bone marrow aspiration (RT–PCR assay) and determination of serum PSA concentrations in predicting bone involvement in the patients recruited for this study. The sensitivity and specificity for a serum PSA concentration of 25 ng $ml^{-1}$ or greater were 87.5% and 89.5% respectively. The positive predictive value was 87.5%, and the lower and upper 95% confidence limits were 71.3% and 100% respectively. The negative predictive value was 89.5%, with the 95% confidence limits being 75.7% (lower) and 100% (upper). The sensitivity and negative predictive value were higher than those of bone scan and bone marrow aspiration in predicting metastatic bone diseases.

### DISCUSSION

We developed the RT–PCR assay to detect metastatic prostate cancer cells (Deguchi et al, 1993). The RT–PCR assay detects PSA mRNA expression from mRNA corresponding to 0.1 LNCaP prostate cancer cells and a single LNCaP cell mixed in $10^6$ peripheral blood mononuclear cells. This assay has a higher sensitivity than other conventional microscopic or immunocytochemical techniques to detect prostate cancer cells in lymph nodes. In this study, 35 bone marrow specimens obtained from previously untreated prostate cancer patients were examined for expression of PSA mRNA using this RT–PCR assay. In 7 of 26 patients with negative bone scans, the RT–PCR assay detected PSA mRNA expression in the bone marrow. Of seven patients with positive RT–PCR assays, five patients had locally extensive prostate cancer, but two patients had clinically or histologically localized prostate cancer. In previous studies, immunocytochemistry detected micrometastatic prostate cancer cells in the bone marrow of 19.5% of the patients with localized prostate cancer (Oberneder et al, 1994), and another RT–PCR assay, specific for PSA mRNA, also found occult bone marrow micrometastases in 20% of patients with pathologically confined prostate cancer (Wood et al, 1994*b*). These findings together with the results of our study demonstrate that micrometastases are present in the bone marrow of patients with clinically and/or pathologically localized prostate cancer. Although not yet conclusive, a small number of PSA-expressing cells detected by RT–PCR in the bone marrow could constitute clinically significant bone metastases. The detection of micrometastatic cells in the bone marrow of patients with operable breast, gastrointestinal and non-small-cell lung cancer is a prognostic indicator of early relapse (Cote et al, 1991; Schlimok et al, 1991; Lindenmann et al, 1992; Pantel et al, 1993). Therefore, in patients with prostate cancer, the detection of PSA mRNA expression using RT–PCR should be useful in determining the possibility of curative surgery or in identifying patients at risk of metastatic diseases.

As demonstrated in this study, the largest concern regarding the clinical use of this method is its site-specific sensitivity. Of nine patients with positive bone scans, four patients had no expression of PSA mRNA in the bone marrow detected by RT–PCR. In these four patients, metastases were found in a few ribs and/or vertebrae without involvement of the pelvic bones, compared with the five patients with positive RT–PCR that had extensive metastases on bone scans found in multiple bones including the pelvic bones. Immunocytochemistry detected metastatic prostate cancer cells in the bone marrow of 57.1–100% of the patients with overt bone metastases (Mansi et al, 1988; Bretton et al, 1994; Oberneder et al, 1994). Another RT–PCR assay detected PSA mRNA in all five patients with metastatic diseases (Wood et al, 1994*b*). That assay detected a single LNCaP cell out of $10^6$ cells of peripheral blood mononuclear cells with a sensitivity equal to our assay (Deguchi et al, 1993; Wood et al, 1994*a*). In the previous reports, information concerning the extent of metastatic disease or the presence or absence of pelvic bone metastases was not available, but two or more aspirates were taken from various sites of the iliac crests and/or the sternum for immunocytochemistry or RT–PCR (Mansi et al, 1988; Bretton et al, 1994; Wood et al, 1994*b*). In our study, only one bone marrow aspirate was obtained from one site of the iliac crest for RT–PCR. Therefore, to facilitate the clinical utility of bone marrow aspiration for the detection of micrometastases, multiple sampling of bone marrow from various sites will be needed. Otherwise, a more sensitive RT–PCR assay involving nested PCR strategies may be required to increase the sensitivity (Israeli et al, 1994; Eschwege et al, 1995).

A statistically significant difference was observed in the distribution of serum PSA concentrations between patients with positive bone scan findings and those with negative findings. In patients with negative bone scans, a significant difference was also found between patients with positive RT–PCR assays and those with negative assays. These findings were in concordance with a previous

© Cancer Research Campaign 1997

bone micrometastasis study using RT–PCR (Wood et al, 1994*a*). Recently, the negative predictive value (99.7%) of a low serum PSA concentration for bone scan findings has been demonstrated (Chybowski et al, 1991), but an elevated serum PSA concentration has not been predictive of bone metastases in individual cases (Osterling 1991; Kleer et al, 1993). However, this study has demonstrated that patients with a serum PSA concentration of 25 ng $ml^{-1}$ or greater had a significantly higher probability of bone involvement and that the sensitivity and negative predictive value for a serum PSA concentration of 25 ng $ml^{-1}$ or greater in predicting metastatic bone diseases were higher than those of bone scan and bone marrow aspiration. Therefore, these findings suggest that a high serum PSA concentration might be the most useful indicator of bone involvement, in clinical terms, for patients with prostate cancer. Methods such as RT–PCR which have the ability to detect a tumour burden, including micrometastases, will facilitate the establishment of a precise correlation between serum PSA concentration and clinical stage.

Limitations of our study include the small sample size and insufficient information about the clinical significance of bone marrow micrometastases. Further studies with a large population and with long-term follow-up of patients with bone marrow micrometastases are needed. Nevertheless, this study suggests that RT–PCR could potentially develop into a relevant tool for the assessment of micrometastases to the bone and establish a precise correlation between serum PSA concentration and bone involvement in patients with prostate cancer.

### REFERENCES

Bretton PR, Melamed MR, Fair WR and Cote RJ (1994) Detection of occult micrometastases in the bone marrow of patients with prostate carcinoma. *Prostate* **25**: 108–114

Chybowski FM, Larson Keller JJ, Bergstralh EJ and Oesterling JE (1991) Predicting radionuclide bone scan findings in patients with newly diagnosed, untreated prostate cancer: prostate specific antigen is superior to all other clinical parameters. *J Urol* **145**: 313–318

Cote RJ, Rosen PP, Lesser ML, Old LJ and Osborne MP (1991) Prediction of early relapse in patients with operable breast cancer by detection of occult bone marrow micrometastases. *J Clin Oncol* **9**: 1749–1756

Deguchi T, Kuriyama M, Shinoda I, Okanao M, Ban Y, Matsui H, Yamada A, Saito I and Kawada Y (1991) Immunological comparison between prostate-specific antigen and γ-seminoprotein. *Urol Res* **19**: 25–30

Deguchi T, Doi T, Ehara H, Ito S, Takahashi Y, Nishino Y, Fujihiro S, Kawamura T, Komeda H, Horie M, Kaji H, Shimokawa K, Tanaka T and Kawada Y (1993) Detection of micrometastatic prostate cancer cells in lymph nodes by reverse transcriptase-polymerase chain reaction. *Cancer Res* **53**: 5350–5354

Eschwege P, Dumas F, Blanchet P, LE Maire V, Benoit G, Jardin A, Lacour B and Loric S (1995) Haematogenous dissemination of prostatic epithelial cells during radical prostatectomy. *Lancet* **346**: 1528–1530

Fair WR, Aprikian A, Sogani P, Reuter V and Whitmore WFJr (1993) The role of neoadjuvant hormonal manipulation in localized prostatic cancer. *Cancer* **71** (suppl.): 1031–1038

Israeli RS, Miller WHJr, Su SL, Powell T, Fair WR, Samadu DS, Huryk RF, Deblasio A, Edwards ET, Wise GJ and Heston WDW (1994) Sensitive nested reverse transcription polymerase chain reaction detection of circulating prostatic tumor cells: comparison of prostate-specific membrane antigen and prostate-specific antigen-based assays. *Cancer Res* **54**: 6306–6310

Katz AE, Seaman E, Olsson CA, Otoole KM, Raffo AJ, McMahon D, Cama C, Benson MC, Perlma H and Buttyan R (1994) Molecular staging of prostate cancer with the use of an enhanced reverse transcriptase-PCR assay. *Urology* **43**: 765–775

Kleer E, Larson Keller JJ, Zincke H and Osterling JE (1993) Ability of preoperative serum prostate-specific antigen value to predict pathologic stage and DNA ploidy. *Urology* **41**: 207–216

Kuriyama M, Esaki N, Shinoda I, Ito S, Yamada S, Tokuyama K, Deguchi T, Takahashi Y and Kawada Y (1993) Measurement of serum PA values by a newly developed enzyme immunoassay. *Jpn J Urol* **84**: 244–250

Kwok S (1990) Procedures to minimize PCR product carry-over. In *PCR Protocols: A Guide to Methods and Applications*, Innis MA, Gelfand DH, Sminsky JJ and White TJ (eds), pp. 142–145. Academic press: San Diego.

Lerner SP, Seale-Hawkins C, Caelton CE Jr and Scardino PT (1991) The risk of dying of prostate cancer in patients with clinically localized disease. *J Urol* **146**: 1040–1045

Lindenmann F, Schlimok G, Dirschedl P and Riethmuller G (1992) Prognostic significance of micrometastatic tumour cells in bone marrow of colorectal cancer patients. *Lancet* **340**: 685–689

Mansi JL, Berger U, Wilson P, Shearer R and Coombes RC (1988) Detection of tumor cells in bone marrow of patients with prostatic carcinoma by immunocytochemical techniques. *J Urol* **139**: 545–548

Moreno JG, Croce CM, Fischer R, Monne M, Vihko P, Mulholland G and Gomella LG (1992) Detection of hematogenous micrometastasis in patients with prostate cancer. *Cancer Res* **52**: 6110–6112

Oberneder R, Riesenberg R, Kriegmair M, Bitzer U, Klammert R, Schneede P, Hofstetter A, Riethmuller G and Pantel K (1994) Immunocytochemical detection and phenotypic characterization of micrometastatic tumour cells in bone marrow of patients with prostate cancer. *Urol Res* **22**: 3–8

Osterling JE (1991) Prostate specific antigen: a critical assessment of the most useful tumor marker for adenocarcinoma of the prostate. *J Urol* **145**: 907–923

Pantel K, Izbicki JR, Angstwurm M, Braun S, Passlick B, Karg O, Thetter O and Riethmuller G (1993) Immunological detection of bone marrow micrometastasis in operable non-small cell lung cancer. *Cancer Res* **53**: 1027–1031

Schellhammer PF (1988) Radical prostatectomy: patterns of local failure and survival in 67 patients. *Urology* **31**: 191–197

Schlimok G, Funke I, Pantel K, Strobel F, Lindenmann F, Witte J and Riethmuller G (1991) Micrometastatic tumor cells in bone marrow of patients with gastric cancer: methodological aspects of detection and prognostic significance. *Eur J Cancer* **27**: 1461–1465

Schröder FH, Hermanek P, Denis L, Fair WR, Gospodarowicz MK and Pavone-Macaluso M (1992) The TNM classification of prostate cancer. *Prostate* **4** (suppl.): 129–138

Wood DPJr, Banks ER, Humphreys S and Rangnekar VM (1994*a*) Sensitivity of immunohistochemistry and polymerase chain reaction in detecting prostate cancer cells in bone marrow. *J Histochem Cytochem* **42**: 505–511

Wood DPJr, Banks ER, Humphreys S, McRoberts JW and Rangnekar VM (1994*b*) Identification of bone marrow micrometastases in patients with prostate cancer. *Cancer* **74**: 2533–2540

© Cancer Research Campaign 1997